# Proteomic analysis of age-dependent changes in protein solubility identifies genes that modulate lifespan

**DOI:** 10.1111/j.1474-9726.2011.00765.x

**Published:** 2012-02

**Authors:** Pedro Reis-Rodrigues, Gregg Czerwieniec, Theodore W Peters, Uday S Evani, Silvestre Alavez, Emily A Gaman, Maithili Vantipalli, Sean D Mooney, Bradford W Gibson, Gordon J Lithgow, Robert E Hughes

**Affiliations:** The Interdisciplinary Research Consortium on Geroscience, The Buck Institute for Research on Aging8001 Redwood Blvd., Novato, CA 94949, USA

**Keywords:** *C. elegans*, lifespan, aging, protein solubility, protein aggregation

## Abstract

While it is generally recognized that misfolding of specific proteins can cause late-onset disease, the contribution of protein aggregation to the normal aging process is less well understood. To address this issue, a mass spectrometry-based proteomic analysis was performed to identify proteins that adopt sodium dodecyl sulfate (SDS)-insoluble conformations during aging in *Caenorhabditis elegans*. SDS-insoluble proteins extracted from young and aged *C. elegans* were chemically labeled by isobaric tagging for relative and absolute quantification (iTRAQ) and identified by liquid chromatography and mass spectrometry. Two hundred and three proteins were identified as being significantly enriched in an SDS-insoluble fraction in aged nematodes and were largely absent from a similar protein fraction in young nematodes. The SDS-insoluble fraction in aged animals contains a diverse range of proteins including a large number of ribosomal proteins. Gene ontology analysis revealed highly significant enrichments for energy production and translation functions. Expression of genes encoding insoluble proteins observed in aged nematodes was knocked down using RNAi, and effects on lifespan were measured. 41% of genes tested were shown to extend lifespan after RNAi treatment, compared with 18% in a control group of genes. These data indicate that genes encoding proteins that become insoluble with age are enriched for modifiers of lifespan. This demonstrates that proteomic approaches can be used to identify genes that modify lifespan. Finally, these observations indicate that the accumulation of insoluble proteins with diverse functions may be a general feature of aging.

## Introduction

Longevity and aging phenotypes are under the influence of a large number of functionally diverse genes. Genes modulating lifespan are likely to affect multiple pathways and in many cases exert general effects on cellular homeostasis. Longevity of the nematode *Caenorhabditis elegans* is influenced by hundreds of genes encoding a range of signaling and metabolic processes including an insulin-like signaling pathway that regulates the FOXO transcription factor DAF-16 (Kenyon *et al.*, 1993; Kimura *et al.*, 1997; Ogg *et al.*, 1997). Together with the stress response transcription factor, heat shock factor 1 (HSF-1), DAF-16 regulates the formation protein aggregates in *C. elegans* and also extend lifespan (Kenyon *et al.*, 1993; Lin *et al.*, 1997; Henderson & Johnson, 2001; Hsu *et al.*, 2003; Cohen *et al.*, 2006). Many of the targets of these transcription factors, such as the heat shock proteins, help maintain other proteins in functional states or target them for degradation. Over-expression of even one HSP-encoding gene was sufficient to confer stress resistance and lifespan extension in *C. elegans* and *Drosophila melanogaster* (Yokoyama *et al.*, 2002; Walker & Lithgow, 2003; Morrow *et al.*, 2004; Wang *et al.*, 2004). This suggested that the maintenance of protein conformation is required for lifespan extension and that a loss of protein homeostasis could contribute to aging.

Protein homeostasis plays an important role in a range of age-related diseases, and a number of toxic conformations of mutant human proteins are associated with disease pathology (Balch *et al.*, 2008). When genes encoding such proteins are expressed in invertebrate models, the proteins often form insoluble aggregates that are associated with cellular toxicity and tissue dysfunction. For example, the expansion of polyglutamine (polyQ) residues in various proteins is associated with neurodegenerative disease in humans such as Huntington’s disease and the spinocerebellar ataxias. Toxic effects of expressing mutant polyQ-containing proteins have been observed in model organisms as well. For example, when expanded polyQ proteins are expressed in *C. elegans*, a generalized impairment of protein homeostasis is observed (Satyal *et al.*, 2000; Morley *et al.*, 2002). Likewise, when the A_3-42_β-amyloid peptide (Aβ) is expressed in *C. elegans* muscle, Aβ aggregates form and the worms become paralyzed (Link, 1995; Drake *et al.*, 2003; McColl *et al.*, 2009). Although protein aggregation is well studied in the context of neurodegenerative diseases, its contribution to normal aging is poorly defined. However, models of protein aggregation have been studied during normal aging (Ben-Zvi *et al.*, 2009). It is notable that expression of proteins prone to aggregation accelerated the aggregation of other proteins, highlighting the limiting capacities of the protein homeostasis machinery (Morimoto, 2008). It has been hypothesized that a loss in protein homeostasis during aging may lead to impaired protein solubility and cellular dysfunction (Ben-Zvi *et al.*, 2009).

As modifying expression of genes encoding proteins linked to protein homeostasis can modulate lifespan in the context of age-related disease (Morimoto, 2008), we tested whether global changes in protein conformation could influence lifespan in the context of normal aging (Morimoto & Cuervo, 2009). Using an unbiased biochemical and proteomic approach, we determined which proteins exhibit altered solubility during aging. We have made a comparative quantitative analysis of SDS-insoluble protein extracts from young and old adult *C. elegans* hermaphrodites and have identified a wide range of insoluble proteins that are significantly enriched in older nematodes. When we reduce the expression of a cohort of these individual proteins by RNA interference (RNAi), we frequently observe an extended lifespan. These results are consistent with a model in which an age-related decline in protein homeostasis results in protein conformational change and a shortened lifespan. It further indicates that reduction in levels of a large number of specific insoluble proteins can extend lifespan.

## Results

### Insoluble proteins accumulate in aged *Caenorhabditis elegans*

**Fig. 1:**
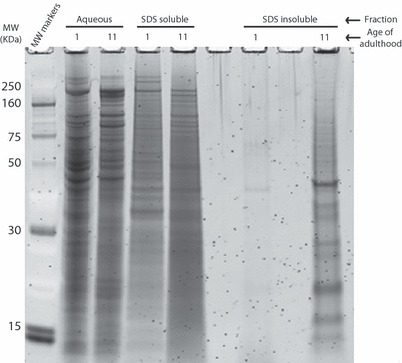
Accumulation of insoluble proteins in aged *Caenorhabditis elegans*. SDS-PAGE of the aqueous, SDS-soluble and SDS-insoluble fractions of 1 and 11-day-old adult animals. Levels of SDS-insoluble proteins are greatly enhanced in extracts from old animals as compared with extracts from young animals.

Protein aggregation can be defined as an alteration in protein quaternary structure leading to loss of solubility. In some cases, this change in can lead to the formation of aggregates that become insoluble in strong detergents such as SDS. We reasoned that if endogenous proteins were becoming insoluble and aggregating during aging, they could be extracted by a solubility based fractionation. This idea was tested using *C. elegans* as a model organism where formic acid (FA) has been used previously to solubilize an aggregation-prone form of GFP (Link *et al.*, 2006). To prevent any transgenerational contamination by self-progeny production, a temperature sensitive sterile strain [TJ1060 *spe-9(hc88)I; fer-15(b26)II*] (Fabian & Johnson, 1994) was used. Synchronous populations of eggs were prepared by mild hypochlorite treatment of gravid adults grown at 20 °C, counted. and allowed to hatch in the absence of an *E. coli* food source. The worms were arrested as first stage larvae (L1s) and thereby were developmentally synchronized. Samples of about 120 mg (wet weight) of worms were collected at the first and eleventh day of adulthood for three biological replicates. We extracted protein by sonication on ice, and postdebris supernatant fractions were normalized for total protein concentration. Samples were then washed several times with an aqueous buffer to remove soluble protein. The water insoluble fraction was resuspended in a similar buffer with 1% SDS and washed several times. The SDS-insoluble fraction was then resuspended in 70% FA for 1 h with vigorous shaking. The aqueous, SDS-soluble, and SDS-insoluble (FA solubilized) fractions were then analyzed by SDS-PAGE. Although the amount of protein solubilized in the aqueous or detergent buffers were similar between young and old worms, FA treatment of the SDS-insoluble fraction yielded significantly more proteins from old worms when compared with young adults (Fig. 1). This result confirmed the hypothesis that SDS-insoluble proteins accumulate during aging in *C. elegans*.

**Fig. 2:**
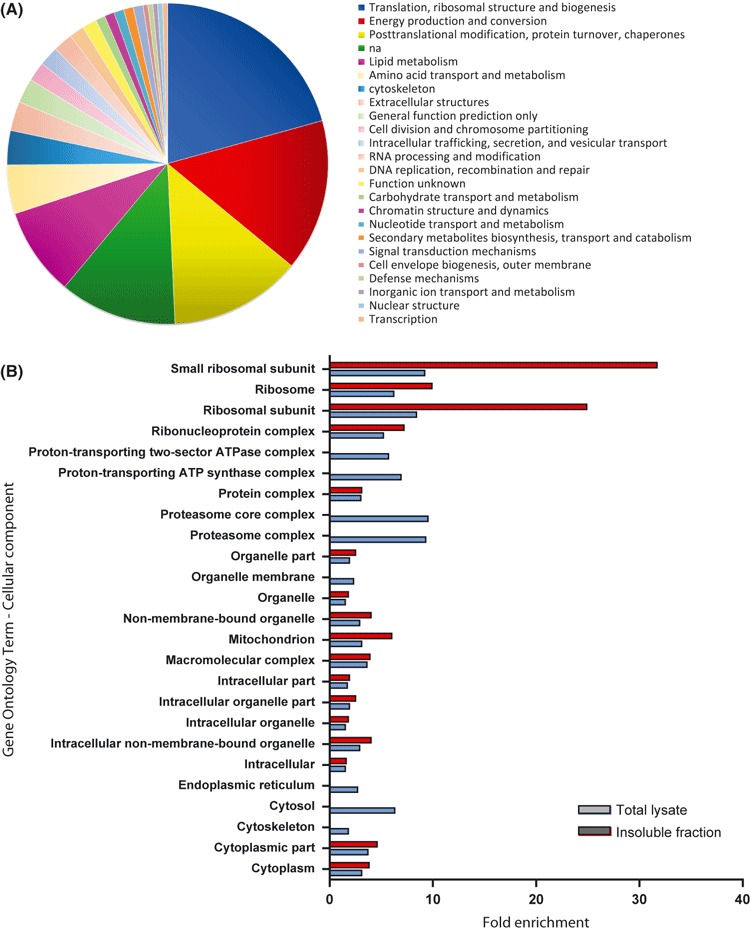
Gene ontology (GO) analysis of proteins observed in the insoluble fraction of old *Caenorhabditis elegans*. A) Functional categories of proteins identified in the insoluble fraction of old worms. Classification is as assigned by Klusters of Orthologous Groups (KOG). B) GO annotations compared between total lysate and the insoluble fraction. Fold enrichment for GO cellular component terms for each of the two fractions is shown.

To identify the insoluble proteins that accumulate in old worms, we first fractionated the protein extracts from three replicate sets of young adults and old worms each as described earlier to obtain a total of six SDS-insoluble fractions. These fractions were then solubilized by FA treatment, dried, and resuspended in TEAB buffer for trypsin digestion. After proteolysis, the resulting peptide fractions from the six protein pools were differentially labeled using six different isobaric amine-specific iTRAQ reagents, mixed, and then fractioned by SCX prior to HPLC separation and mass spectrometry analysis. Under ESI-MS/MS conditions, the iTRAQ chemical tag yields a distinct chemical reporter in the low mass region that can be used as a measure of relative abundance. Therefore, informatic analysis of the tandem spectra from these experiments identified both the peptides (and proteins) that make up these SDS-insoluble fractions, as well as providing a means to determine the relative concentrations among the six protein pools. Using this method, we identified 203 insoluble proteins that are at least 2-fold enriched in aged insoluble fractions when compared with the equivalent fraction prepared form young worms (Fig. 2, Table S1). Proteins with functions related to translation, energy production, and protein homeostasis comprised nearly 50% of the proteins in the insoluble fraction (Fig. 2, Table S1). Western blot analysis against aconitase-2 and DAF-21 independently confirmed the mass spectrometry results by showing an accumulation in the insoluble fraction of old animals (Fig. S1).

To assess the nature of this SDS-insoluble fraction relative to the total proteome of *C. elegans*, we carried out gene ontology (GO) analysis to determine which annotation categories were significantly enriched among proteins in this fraction. As the list of insoluble proteins was obtained through a biochemical fractionation, we reasoned that we should compare it with a total lysate. To do this, we also carried out GO analysis of a list of around 1200 proteins identified by iTRAQ in a total lysate (Fig. 2). Although it was clear that proteins of diverse predicted functions were represented in the insoluble fraction, there was notable representation of some important functional groups. We found highly significant enrichments of proteins associated with ribonucleoprotein complexes, particularly components of the small ribosomal subunit (Fig. 2).

### Many proteins that become insoluble during aging also determine lifespan

**Fig. 3:**
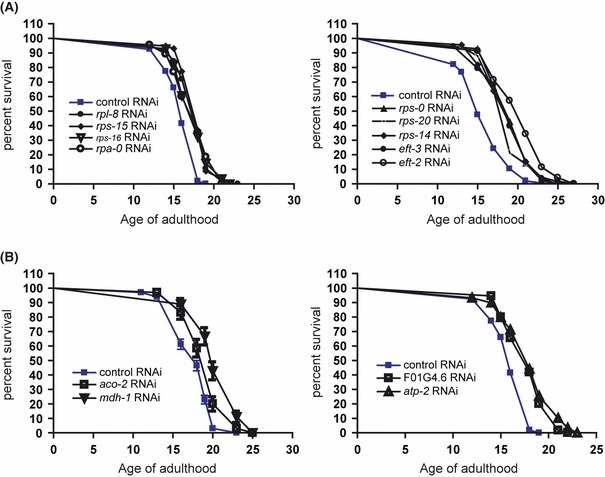
A large fraction of the insoluble proteins reduce *Caenorhabditis elegans* lifespan. Synchronous populations of adult worms were subjected to RNAi knock-down of transcript levels of various genes encoding proteins identified in the insoluble fraction of old worms. Kaplan-Meyer survival curves were generated based upon survival at the indicated time points. A) Survival curves while knocking down transcript levels of genes encoding translation-related proteins. B) Survival curves while knocking down transcript levels of genes encoding mitochondrial proteins.

**Fig. 4:**
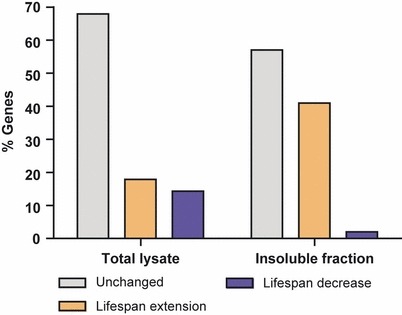
The SDS-insoluble fraction is highly enriched for proteins that negatively modulate lifespan. Comparison of survival data after knock-down of genes selected from a total lysate and the insoluble fraction. Percentage of genes with no effect, extending, and decreasing lifespan is shown.

To identify proteins among the 203 proteins in the SDS-insoluble fraction that might influence lifespan, the top 99 genes with the largest fold-change between young and old worms were selected for reduction in gene expression by RNA interference and survival analysis. We began RNAi treatment only after the animals reached the late L4 stage to avoid developmental effects. Each RNAi treatment that extended lifespan was tested at least three times. Strikingly, reducing the expression of 41% of the insoluble proteins significantly extended mean adult hermaphrodite lifespan (Fig. 3, Tables 1 and S2). Lifespan extensions varied between a few percent up to 30%. The lifespan extension is not attributable to delays in development as the worms reached adulthood synchronously with control (RNAi vector only) worms. Notably, only two of the genes tested from this group caused a decrease in lifespan upon knock-down. We then determined whether the insoluble protein cohort was enriched for functions that limit lifespan under our assay conditions. To test this, we randomly selected 28 proteins previously identified in a total lysate and selectively knocked down transcript levels of the respective genes. Survival analysis revealed that 18% of the RNAi treatments extended lifespan and 14% reduced it (Fig. 4, Table S3). Taken together, our survival data show that by fractionating a total lysate according to solubility, there is a significant enrichment for proteins that contribute to a normally short lifespan in an SDS-insoluble fraction.

**Table 1:** Summary of survival analysis knocking down transcripts encoding insoluble proteins

Gene	ORF	Accession number*	Description	Mean extension (%)†	*P* value	*N*
*rps-16*	T01C3.6	Q22054	Protein of the small ribosomal subunit	8.3	0.0012	75
*aco-2*	F54H12.1	P34455	Probable mitochondrial aconitase	20.5	<0.0001	82
*rps-15*	F36A2.6	Q9XVP0	Protein of the small ribosomal subunit	9.7	0.0017	72
*atp-2*	C34E10.6	P46561	Mitochondrial membrane ATP synthase	14.8	<0.0001	76
C34B2.6	C34B2.6	O44952	Mitochondrial Lon protease ortholog	10.8	<0.0001	85
*his-37*	C50F4.7		H4 histone	4.5	0.0316	77
F23B12.5	F23B12.5	Q19749	Component of mitochondrial pyruvate dehydrogenase	9.9	<0.0001	62
F01G4.6	F01G4.6	Q0PDK0	Probable mitochondrial phosphate carrier protein	11.9	<0.0001	74
*inf-1*	F57B9.6	P27639	Eukaryotic initiation factor 4A	21.4	<0.0001	73
F56D2.1	F56D2.1	P98080	Mitochondrial cytochrome b-c1 subunit	14.1	<0.0001	79
*rpl-8*	Y24D9A.4	Q9XVF7	Protein of the large ribosomal subunit	12.3	<0.0001	77
*rps-5*	T05E11.1	P49041	Protein of the small ribosomal subunit	4.0	0.0037	62
*pyc-1*	D2023.2	O17732	Pyruvate carboxylase-1	4.9	0.0043	72
*mdh-1*	F20H11.3	O02640	Malate dehydrogenase-1	4.1	0.0443	73
*eft-3*	F31E3.5	P53013	Elongation factor 1-alpha	18.1	<0.0001	68
*rps-0*	B0393.1	P46769	Protein of the small ribosomal subunit	15.1	<0.0001	60
*eft-2*	F25H5.4	P29691	Elongation factor 2	7.1	0.0052	62
*tbb-2*	C36E8.5	P52275	Beta tubulin ortholog	5.1	0.0485	67
*rpa-0*	F25H2.10	Q93572	Protein of the large ribosomal subunit	6.6	<0.0001	74
*rpl-23*	B0336.10	P48158	Protein of the large ribosomal subunit	17.7	<0.0001	71
*rps-20*	Y105E8A.16	Q8WQA8	Protein of the small ribosomal subunit	23.6	<0.0001	62
*rpl-31*	W09C5.6	Q9U332	Protein of the large ribosomal subunit	15.5	<0.0001	58
F09F7.4	F09F7.4	Q19278	Probable Enoyl-CoA hydratase	10.7	0.0005	57
*rpl-10a*	Y71F9AL.13	Q9N4I4	Protein of the large ribosomal subunit	23.2	<0.0001	65
ZK829.4	ZK829.4	Q23621	Probable glutamate dehydrogenase	11.1	<0.0001	62
*rps-14*	F37C12.9	P48150	Protein of the small ribosomal subunit	6.4	0.0197	58
*cpn-3*	F28H1.2	O01542	Calponin ortholog	9.9	0.0027	61
B0250.5	B0250.5	Q9XTI0	Probable mitochondrial 3-hydroxyisobutyrate dehydrogenase	10.2	0.0005	61
*rrt-1*	F26F4.10	Q19825	Probable arginyl-tRNA synthetase	26.6	<0.0001	33
*rpl-10*	F10B5.1	Q09533	Protein of the large ribosomal subunit	20.4	<0.0001	67
*rpl-7*	F53G12.10	O01802	Protein of the large ribosomal subunit	9.5	0.001	62
*cct-1*	T05C12.7	P41988	Alpha subunit of cytosolic chaperonin complex	10.1	0.0004	69
*rpl-17*	Y48G8AL.8	Q9BL19	Protein of the large ribosomal subunit	13.2	<0.0001	51
*acs-4*	F37C12.7	Q20121	Probable fatty acid Co-A synthetase	6.1	0.0334	56
*rps-11*	F40F11.1	Q20206	Protein of the small ribosomal subunit	29.8	<0.0001	36
*rpl-3*	F13B10.2	Q6BEU5	Protein of the large ribosomal subunit	28.6	<0.0001	64
*rps-23*	F28D1.7	Q19877	Protein of the small ribosomal subunit	22.7	<0.0001	60
*rack-1*	K04D7.1	Q21215	Probable guanine nucleotide binding protein subunit beta-2-like 1	9.8	0.0046	37
*pab-1*	Y106G6H.2	Q9U302	Ortholog of receptor activated c kinase	24.0	<0.0001	64
*cct-8*	Y55F3AR.3	Q9N358	Theta subunit of cytosolic chaperonin complex	16.0	<0.0001	60
*sca-1*	K11D9.2	Q9XU13	Sarco-endoplasmic reticulum Ca^2+^ ATPase	18.6	<0.0001	58

Lifespan experiments were repeated three independent times. Lifespan extension upon RNAi treatment was considered when a significant (*P* < 0.05) extension was observed in all three experiments. Representative data for one experiment are shown. *P-*values for each individual experiment are shown.

* UniProtKB protein accession number.

† Relative to lifespan of wild-type feeding on control RNAi strain L4440.

## Discussion

Aging is a major risk factor for a wide range of diseases including cancer, diabetes, and neurodegeneration. The accumulation of insoluble proteinaceous aggregates is a shared feature in the most common neurodegenerative diseases (Alzheimer’s, Parkinson’s, Huntington’s and amyotrophic lateral sclerosis), and these have been sometimes referred to as proteinopathies. We hypothesized that processes related to protein aggregation occurring during normal aging (in the absence of disease) may share features with pathologic processes underlying late-onset neurodegeneration. We thus considered a biochemical characterization of protein homeostasis and protein aggregation during normal aging important in understanding the cellular pathology of late-onset neurodegeneration.

Using an unbiased approach, we have identified which proteins share biochemical characteristics of known aggregation-prone proteins implicated in neurodegeneration and critically whether these proteins have any connection to normal aging. We set out to identify proteins that exhibited age-dependent changes solubility in adult *C. elegans.* By analysis and comparison of protein extracts from young and old worms, we were able to quantify the relative abundance of SDS-insoluble proteins in each sample thus generating a sample of the proteins that become SDS insoluble during normal aging. We found that no one protein or class of proteins were represented in the insoluble protein of older worms. Indeed a wide variety of biochemical and physiological functions are implied by homology across 203 proteins identified. However, there is a notable enrichment for proteins encoding ribosomal subunits.

Using similar methods, David *et al.* (2010) reported a wide range of insoluble proteins in aged *C. elegans*. They indicate that across four independent experiments, 711 proteins were identified with high confidence as being enriched in the insoluble fraction of aged animals. Of these 711 proteins, they identified 461 proteins that increased in all four independent experiments. Comparison of our list of proteins that were enriched in the insoluble fraction in aged animals to those generated by David *et al.* reveals a significant overlap in protein identities; over half of the proteins identified in our study (107 of 203) were present in the age-dependent insoluble set of 461 proteins identified by David *et al.*, while nearly 80% of the proteins (162 of 203) on our list were seen in at least one of their experiments as being enriched in aged worms. Unlike the report from David *et al.,* we found very little SDS-insoluble protein in young animals as analyzed by PAGE (Fig. 1) or iTRAQ mass spectrometry (Table S1), and nearly every protein that was identified (203 of 207 proteins) increased in aged samples by at least 2-fold. These discrepancies are likely due to slightly different preparation protocols. Our separation protocol seems to be more stringent in identifying SDS-insoluble proteins that accumulate with age. However, the significant overlap between the data sets indicates that both studies purified a similar fraction of the proteome.

Given this significant overlap of protein identities, it is not surprising that we find similar biological processes enriched after GO analysis [NIH-DAVID gene ontology (Huang da *et al.*, 2009)]. Like David *et al.*, we find a significant enrichment in the biological processes of embryonic development, translation, growth, cofactor metabolic process, and determination of adult lifespan. Both studies also identified a significant enrichment in proteins localized to the ribonucleoprotein cellular component. However, our dataset was not significantly enriched in either the chaperone or proteasome cellular component GO categories. Instead, our dataset was significantly enriched in proteins localized to the mitochondrion (Fig. 2). Given the differences in insoluble protein preparation, it is possible that the cellular component GO categories enriched in the David *et al.* dataset represent a broader spectrum of proteins that become insoluble with age while the enriched cellular component GO categories identified in our dataset represent the ‘core’ protein components that become insoluble with age.

We went on to evaluate the effects of reducing expression of genes encoding insoluble proteins in lifespan and were struck by the discovery that nearly half of the tested proteins were limiting lifespan in that we observed an increased lifespan when their abundance is reduced. We compared this with a randomly selected gene set where only 18% of the RNAi treatments extended mean lifespan and 14% of the RNAi reduced lifespan. In contrast, after fractionation by solubility, only 2% of the tested genes negatively affected lifespan. Taken together, these data suggest that insoluble proteins contribute to the relatively short lifespan of wild-type *C. elegans.* It is possible that lifespan is extended by distinct mechanisms for each protein but as these proteins were identified using a biochemical approach applied to prepare toxic, disease-associated proteins, it is also possible that worm proteins share structural features associated with toxicity such as an ability to interfere with protein homeostatic machinery.

The notion that lifespan is limited by the capacity of the protein homeostatic network is supported by a series of genetic studies in which the expression of components of the network has been manipulated. In *C. elegans* and *Drosophila,* over-expression of molecular chaperone genes and their transcriptional regulator HSF-1 led to lifespan extension whereas lowering their expression suppressed longevity (Garigan *et al.*, 2002; Hsu *et al.*, 2003; Walker & Lithgow, 2003; Morley & Morimoto, 2004; Morrow *et al.*, 2004; Wang *et al.*, 2004). Furthermore, mutation of *age-1* not only extends lifespan but also resulted in the suppression of protein aggregation in *C. elegans* expressing polyglutamine (polyQ), suggesting a link between the mechanisms that determine lifespan and protein aggregation rates (Morley *et al.*, 2002; Cohen *et al.*, 2006). As *age-1* regulates the levels of molecular chaperones (Walker *et al.*, 2001; Hsu *et al.*, 2003), the suppression of protein aggregation and lifespan extension could result of an enhanced protein homeostasis capacity. This model is also consistent with observations that small molecules that suppress protein aggregation also extend lifespan (Alavez *et al.*, 2011).

We have considered whether other mechanisms are at play in our lifespan. Some functions associated with proteins from the insoluble fraction have been previously implicated in limiting lifespan. For example, in a number of model organisms, mutations in genes encoding translation factors result in extended lifespan. It is possible that a reduction in bulk translation rates extends lifespan or that there is an increase in the translation of a subset of mRNAs encoding longevity functions (Zid *et al.*, 2009). In our comparative proteomic analysis, we also found an enrichment for translation functions. Our data also agree with previous studies in that we observed increased lifespan after reducing transcript levels of translation-related genes. Perhaps this is the result of altered translation, although the fact these proteins become insoluble during aging suggests an alternative explanation as to why translation factors limit lifespan. It is possible that the aggregation of components of the translational apparatus itself limits lifespan by the demands this places on protein homeostatic mechanisms.

Here, we have demonstrated that aging results in a range of distinct proteins species entering an insoluble state. We demonstrate further that decreasing the abundance of individual proteins in this insoluble compartment results in an increased lifespan, suggesting that these proteins can limit lifespan. The commonality between the behavior of proteins associated with specific age-related diseases and a range of cellular proteins that influence lifespan during normal aging provides a novel context for considering the role of aging as a primary risk factor for the most common forms of neurodegeneration.

## Experimental procedures

### *Caenorhabditis elegans* strains and the generation of transgenic strains

The Bristol N2 strain was used as the wild-type strain and was obtained from the *Caenorhabditis* Genetics Center. TJ1060 strain was a gift from Thomas E. Johnson (Univ. of Colorado, Boulder).

### Insoluble protein extraction

TJ1060 [*spe-9(hc88)I; fer-15(b26)II*] temperature sensitive mutants were grown and aged in synchronous mass cultures (Fabian & Johnson, 1994). After bleaching the worms, L1s were arrested in S-Basal and thereby synchronized. Approximately 5000 animals were plated in each enriched peptone plate spotted with *E. coli* (NA22) and then aged at 25 °C (restrictive temperature). Samples of about 120 mg of worms were collected from several plates at 1 and 11 days of adulthood for three biological replicates. Most of the very few dead worms were stuck to the bacterial lawn when collecting the samples. Worms were washed several times with S-basal and resuspended in aqueous lysis buffer (20 mm Tris base, 100 mm NaCl, 1 mm MgCl_2,_ pH = 7.4) with protease inhibitor cocktail COMPLETE® from Roche Diagnostics (Mannheim, Germany). The samples were sonicated on ice and then centrifuged at 3000 ***g*** to remove carcases. At this point, all samples were normalized for total protein concentration as assessed by BCA assay (Thermo Fisher Scientific, Rockford, IL, USA). Samples were centrifuged at 16 000 ***g*** and washed three times to extract the water-soluble fraction. The pellet was then resuspended and washed three times in the same buffer containing 1% SDS to retain the detergent soluble fraction. The insoluble fraction was then treated for 1 h with 70% FA with vigorous shaking at room temperature. The acidic fractions were dried in a Speed-Vac at 25 °C.

### Isobaric tag for absolute and relative quantitation

#### Preparation for mass spectroscopy

FA treated protein samples were resuspended in equal volumes of solution consisting of 200 mm TEAB, 0.1% SDS, and 1% NP-40 at pH 8.0, to maintain the same volume to total initial protein ratio from *C. elegans*. Samples were able to resuspend over 2 h on ice with occasional vortexing. BCA quantitation was performed, and equal volumes were collected from all six samples (three biological repeats of worms at the first and eleventh day of adulthood) resulting in 50 μg of total protein being collected. This volume was chosen because it resulted in the maximum amount of protein that can be labeled using aliquoted iTRAQ reagents while utilizing the same volume of each sample. Samples were prepared and labeled with iTRAQ 6-plex reagents according to the manufacturer’s guidelines (Applied Biosystems, Foster City, CA, USA), reduced (5 mm tris(2-carboxyethyl) phosphine at 60 °C for 60 min), and blocked (10 mm methyl methanethiosulfonate) for 10 min at RT. Samples were digested at 37 °C overnight with 2 μg trypsin (Promega, Madison, WI, USA), with 1 μg added ∼6 h after digestion began. Samples were then iTRAQ (Applied Biosystems) labeled for 2 h after which all samples were combined into strong cation exchange (SCX) loading buffer (Applied Biosystems) at pH 3.0 quenching the labeling reaction. Combined samples were cleaned-up using a SCX cartridge (Applied Biosystems) and eluted using a high salt elute of 350 mm KCl and 25% CAN (Applied Biosystems). The peptides were then dried in a Speed-Vac at 25 °C.

#### Liquid chromatography and mass spectrometry

Samples were redissolved in 2% ACN (0.1% FA). An estimated 1.0 μg of labeled sample was loaded and desalted on a Dionex Acclaim LC Packings μ-Precolumn Cartridge (5 mm × 300 μm RP-C18 trap column, 5 μm beads, 100 Å pore size). Chromatography was performed on a Dionex Acclaim Pepmap column (75 μm × 150 mm RP-C18, 3 μm beads, 100 Å pore size) with a 2-80% ACN (0.1% FA) 100 min gradient at a flow rate of 300 μL min^−1^ (2–35% ACN in 0–70 min, 35–80% ACN 70–100 min). A QSTAR Elite Q-TOF (Applied Biosystems) operated in a positive ion mode (2.3 kV) performed the analysis, with the five most intense ions between 350 and 1600 *m*/*z* (over 20 counts) and with charge states of 2–5 in the TOF-MS scan were subjected to information-dependent acquisition MS-MS with Smart Exit function employed (setting 8). Ions within ±100 ppm of a former sampled ion were dynamically excluded for 120 s. The sample was analyzed five times on the mass spectrometer as technical replicates.

#### Data processing

The data from five runs were searched and combined using Protein Pilot software 3.0 (Applied Biosystems) with background correction and bias correction turned off. Searching was conducted with SwissProt Database version 2.0 (*Caenorhabditis elegans*: 22 983 entries). Only peptides of confidence >50% were used for protein quantification, and a 95% confidence (unused prot score > 1.3) threshold for protein identification as determined by Protein Pilot 3.0 was the criteria for inclusion into the final protein report.

### Western blots

Extraction of insoluble protein was preformed as described earlier. Total protein levels were measured by BCA assay (Thermo Fisher Scientific), and loading amounts were normalized. SDS-PAGE was performed using 4–12% Bis–Tris gel NuPAGE system (Invitrogen, Carlsbad, CA, USA). DAF-21 antibody was obtained from Enzo Life Sciences (Plymouth Meeting, PA, USA) and Aconitase-2 antibody from Abcam (Cambridge, MA, USA).

### RNA interference

RNA interference was undertaken as previously described (Olsen *et al.*, 2006). Briefly, worms grown at 20 °C were used to lay eggs. Synchronized L4 larvae were moved to RNAi plates (NGM containing 100 μg mL^−1^ of ampicillin, 20 μg mL^−1^ tetracycline, and 1 mm IPTG) spotted with bacteria expressing double stranded RNAi. *hsf-1* RNAi was from the Ahringer library and confirmed by sequencing.

### Adult lifespan assays

Wild-type worms were grown in mass cultures in enriched peptone plates with NA22 as food source (Strange *et al.*, 2007). Eggs were synchronized by bleaching gravid adults (Fabian & Johnson, 1994) and arresting L1 larvae in S-Basal overnight. Worms were then grown in regular NGM plates with OP50 as food source and transferred to RNAi plates (NGM containing 100 μg mL^−1^ of ampicillin, 20 μg mL^−1^ tetracycline, and 1 mm IPTG) at late L4 stage. Most RNAi clones were from Open Biosystems library and when not available the Ahringer library clones were used. All RNAi treatments that extended lifespan were repeated at least three times. Data analysis was carried out using GraphPad Prism 4 (GraphPad Software Inc., San Diego, CA, USA).

### Gene ontology analysis

GO analysis was carried out using NIH DAVID Bioinformatic Database (http://david.abcc.ncifcrf.gov/). The 203 proteins that were identified as being enriched in aged *C. elegans* were queried against the total protein *C. elegans* proteome. Subsequent lists of significantly enriched biological process and cellular component GO categories were filtered to exclude reported GO categories with a false discovery rate >0.05 and parsed of redundant categories.

## Supporting Information

**Fig. S1** DAF-21 and ACO-2 accumulate in theinsoluble fraction of aged *C. elegans*.

**Table S1** List of insoluble proteins in aged *C. elegans*.

**Table S2** Survival analysis knocking down transcripts encoding insoluble proteins

**Table S3** Survival analysis of a randomly generated list of genes encoding proteins identified in a total lysate

As a service to our authors and readers, this journal provides supporting information supplied by the authors. Such materials are peer-reviewed and may be re-organized for online delivery, but are not copy-edited or typeset. Technical support issues arising from supporting information (other than missing files) should be addressed to the authors.
