# Supplementary material for: Proteomic analysis of age-dependent changes in protein solubility identifies genes that modulate lifespan
Source: Aging Cell. 2012 Feb;11(1):120–7. doi: 10.1111/j.1474-9726.2011.00765.x (PMC3437485; doi:10.1111/j.1474-9726.2011.00765.x)
Supplement: Supplementary file 2 [file acel0011-0120-SD2.docx]

**Fig. S1** DAF-21 and ACO-2 accumulate in the insoluble fraction of aged *C. elegans.* Worm samples were collected at 1 and 11 days of adulthood and protein was fractionated by solubility. Immunoblots are shown for aconitase-2 (top) and DAF-21 (bottom) for 1 and 11 day old worms both in a total homogenate and in the insoluble fraction. Actin loading controls are shown.

**Table S1** List of insoluble proteins in aged *C. elegans.*

**Table S2** Survival analysis knocking down transcripts encoding insoluble proteins. Mean lifespan extension values and statistical analysis of Kaplan-Meyer Survival curves generated by knocking down transcript levels of genes encoding proteins that become insoluble with age. Lifespan extension was defined as significant for *p≤*0.05 in all three repeats.

**Table S3** Survival analysis of a randomly generated list of genes encoding proteins identified in a total lysate. Mean lifespan extension values and statistical analysis of Kaplan-Meyer Survival curves generated by knocking down transcript levels of genes encoding proteins identified in a total lysate. Proteins were selected from a list of 1200 using a random number generator. Lifespan extension was defined as significant for *p≤*0.05 in two repeats.
